# Anti-Hyperglycaemic Evaluation of *Buddleia indica* Leaves Using In Vitro, In Vivo and In Silico Studies and Its Correlation with the Major Phytoconstituents

**DOI:** 10.3390/plants10112351

**Published:** 2021-10-30

**Authors:** Sameh S. Elhady, Fadia S. Youssef, Abdulrahman M. Alahdal, Diena M. Almasri, Mohamed L. Ashour

**Affiliations:** 1 Department of Natural Products, Faculty of Pharmacy, King Abdulaziz University, Jeddah 21589, Saudi Arabia; ssahmed@kau.edu.sa; 2 Department of Pharmacognosy, Faculty of Pharmacy, Ain-Shams University, Abbasia, Cairo 11566, Egypt; 3 Department of Pharmacy Practice, Faculty of Pharmacy, King Abdulaziz University, Jeddah 21589, Saudi Arabia; aalahdal2@hotmail.com (A.M.A.); dalmasri@kau.edu.sa (D.M.A.); 4 Department of Pharmaceutical Sciences, Pharmacy Program, Batterjee Medical College, Jeddah 21442, Saudi Arabia

**Keywords:** anti-hyperglycaemic activity, *α*-amylase, *Buddleia indica*, *α*-glucosidase, Scrophulariaceae, streptozotocin-induced diabetes

## Abstract

*Buddleia indica* Lam. is an ornamental evergreen shrub with few reports concerning its phytoconstituents and biological activities. Herein, the antihyperglycaemic activity of *B. indica* leaves methanol extract (BIT) was evaluated for the first time using in vitro and in vivo studies. Molecular modelling was performed for its major phytoconstituents that were further subjected to ADME/TOPAKT (absorption, distribution, metabolism, excretion and toxicity) prediction. BIT revealed considerable reduction in glucose concentration by 9.93% at 50 μg/mL using 3T3-L1 adipocyte culture. It displayed substantial inhibition versus *α*-glucosidase and *α*-amylase with IC_50_ 205.2 and 385.06 μg/mL, respectively. In vivo antihyperglycaemic activity of BIT and the ethyl acetate fraction (BIE) was performed using streptozotocin-induced diabetes in rat model. BIT and BIE effectively ameliorate oxidative stress markers in addition to reducing serum blood glucose by 56.08 and 54.00%, respectively, and are associated with a substantial increase in serum insulin by 4.1 and 12.7%, respectively. This can be attributed to its richness with polyphenolic compounds comprising flavonoids, phenolic acids, phenyl propanoids and iridoids. Molecular docking showed that verbascoside and kaempferol displayed the highest fitting within human *α*-amylase (HA) and human *α*-glucosidase (HG) active sites, respectively. They showed reasonable pharmacokinetic, pharmacodynamic and toxicity properties, as revealed by ADME/TOPKAT study.

## 1. Introduction

Diabetes mellitus is defined as a chronic disease accompanied by plethora of metabolic disorders that occur as a result of either genetic or acquired reasons [1]. Basically, it is characterized by an abnormal high blood glucose level triggered by either reduction in the production of insulin or its resistance. This uncontrolled hyperglycaemia can cause various micro- and macrovascular complications including cardiovascular disorders, neuropathy, nephropathy retinopathy as well as ulceration, with a subsequent high rate of morbidity and mortality worldwide [2,3]. Recently, it has been considered as the third lethal cause after cancer and cardiac diseases in terms of people affected across the globe. The International Diabetes Federation estimates that 592 million people will be affected by diabetes by 2035 [4].

In spite of the great developments in the field of drug discovery and the presence of a large number of synthetic drugs belonging to different classes that effectively counteract hyperglycaemia, the control of diabetes still constitutes a major problem that is undoubtedly due to the pronounced adverse effects of the synthetic compounds [5]. This necessitates the discovery of relatively safer, less expensive new drug entities derived from natural sources compared to synthetic ones which in turn are highly acceptable by a wide category of patients both in developing as well as developed countries [6].

*Buddleia* (*Buddleja*) belonging to Scrophulariaceae includes about 100 species with native habitats in North and South America, Africa, and Asia. Multiple biological activities have been attributed to Genus *Buddleia*, comprising its hepatoprotective, antioxidant, immunosuppressive, analgesic, antihypertensive, and antimicrobial activities. This observed biological potential relied upon its secondary metabolites including flavonoids, iridoid glucosides and phenylpropanoids [7].

*Buddleia indica* Lam. is an ornamental evergreen shrub of African, mainly Madagascan origin. Few reports were found in literature regarding its secondary metabolites and biological activities. This includes the antioxidant, hepatoprotective, antimicrobial and antiviral activities as well as the isolation and identification of some phenolic acids, flavonoids and iridoids from the plant [8,9].

Herein, we aimed to evaluate for the first time the antihyperglycaemic activity of *Buddleia indica* leaves total methanol extract (BIT) using various in vitro assays such as 3T3-L1 adipocytes, *α*-amylase and *α*-glucosidase inhibitory activity. This was consequently followed by an in vivo study of total methanol extract (BIT), as well as the ethyl acetate fraction (BIE) using streptozotocin-induced diabetes in rat models. To further validate the obtained biological activity results, molecular modelling was performed for major phytoconstituents of *Buddleia indica* leaves previously identified by the authors on different enzymes implicated in the incidence and deterioration of hyperglycaemia such as *α*-glucosidase and *α*-amylase enzymes. The major identified compounds were further subjected to ADME/TOPAKT prediction to determine their pharmacokinetic, pharmacodynamic and toxicity properties. This was done in an effort to search for naturally occurring herbal drugs counteracting diabetes having less adverse effects and highly welcomed by people suffering from hyperglycaemia comparable to synthetic drugs.

## 2. Results and Discussion

### 2.1. Biological Evaluation of the Anti-Hyperglycaemic Activity of Buddleia indica Leaves

#### 2.1.1. In Vitro Anti-Hyperglycaemic Evaluation Using 3T3-L1 Adipocyte Culture

Adipose tissues represent the most important sites for glucose uptake postprandially. 3T3-L1 adipocyte culture acts as an in vitro model that simulates the adipose tissue which is utilized to assess the cellular glucose uptake rate stimulated by insulin. Basically, this represents the rate-limiting step in the control of glucose levels that if disturbed can cause insulin resistance resulting in diabetes, particularly type 2 [10,11]. Thus, a 3T3-L1 adipocyte culture was used for in vitro anti-hyperglycaemic evaluation of the total methanol extract of *Buddleia indica* leaves, where 30 and 50 µg/mL represent the utilized concentrations. A comparison with pioglitazone and insulin, as positive controls, was performed at the same concentrations. Results represented in Table 1 revealed that glucose concentrations were reduced from 430.7 and 434.1 mg/dL to 424.0 and 389.2 mg/dL after receiving BIT at concentrations of 30 and 50 μg/mL, respectively. This revealed mild reduction in the glucose concentration estimated by 1.47% after treatment with 30 μg/mL of BIT when compared to the untreated control group; meanwhile, it showed a considerable reduction in the glucose concentration equal to 9.93% upon treatment with 50 μg/mL of BIT. In this aspect, BIT approaches insulin and pioglitazone, particularly at 50 μg/mL, where they exhibited 12.01 and 10.20%, respectively, however they showed 5.85% and 4.09% reduction in glucose concentration at 30 μg/mL.

#### 2.1.2. In Vitro Anti-Hyperglycemic Evaluation Using Inhibition of *α*-Amylase and *α*-Glucosidase Activity Assay

**Figure 1:**
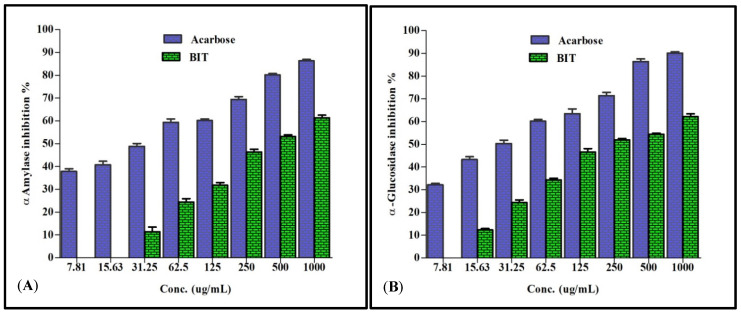
Inhibition% of *Buddleia indica* leaves total methanol extract (BIT) and acarbose at different concentrations versus *α*-amylase (**A**) and *α*-glucosidase (**B**).

*α*-Amylase is a crucial enzyme that helps in the catalysis of the primary steps in the process of converting starch to maltose and subsequently to glucose [12]. Meanwhile *α*-glucosidase is a vital enzyme that acts as a catalyst that effectively helps in the hydrolysis of linear *α*-1,4-linked oligosaccharide substrates with the concomitant production of glucose [13]. These two enzymes represent the main targets in the process of drug discovery aiming to find leads for combating diabetes as well as obesity [2]. Regarding *α*-glucosidase, the total methanol extract of *Buddleia indica* leaves displayed substantial inhibitory potential versus *α*-glucosidase, with IC_50_ estimated by 205.2 μg/mL where acarbose, a standard antihyperglycaemic agent, displayed IC_50_ of 30.52. Concerning *α*-amylase, *Buddleia indica* leaves total methanol extract displayed mild inhibitory potential with IC_50_ estimated by 385.06 μg/mL, where acarbose displayed IC_50_ of 35.02. The inhibition% of *Buddleia indica* leaves total methanol extract (BIT) and acarbose at different concentrations versus *α*-amylase and *α*-glucosidase is displayed in Figure 1A,B.

#### 2.1.3. In Vivo Anti-Hyperglycemic Activity Assessment Using Streptozotocin Induced Hyperglycaemia in Rat

##### Determination of Fasting Blood Glucose and Serum Insulin Levels

Concerning in vivo anti-hyperglycemic activity assessment, STZ was employed to induce hyperglycaemia in animals. It serves as a diabetogenic agent that damages the pancreatic *β*-cell through the massive production of reactive oxygen species (ROS). The entrance of STZ to the pancreatic cells by a glucose transporter (GLUT2) causes severe damage to the DNA via its alkylation that is concomitantly followed by poly ADP-ribosylation. This will be accompanied by depletion of ATP and NAD^+^ within the cells, causing ATP dephosphorylation and affording a substrate for xanthine oxidase action accompanied by the production of hydrogen peroxide, superoxide radicals and hydroxyl ions. In addition, STZ generates large quantities of nitric oxide that prohibits aconitase activity and worsens DNA damage [14]. STZ injection elicited pronounced increase in the levels of fasting blood glucose estimated by 142.61% compared to the normal untreated group (*p* < 0.05), with a concomitant decrease in serum insulin levels by 40.10% relative to the normal group (Table 2). In contrast, oral administration of glibenclamide, GLB, effectively ameliorates hyperglycaemia manifested by a potent decrease of FBG estimated by 46.60% (*p* < 0.05) accompanied by a significant elevation in serum insulin by 37.29% with respect to STZ-induced diabetic animals. Meanwhile, oral treatment of STZ-induced diabetic animals with BIT and BIE effectively reduced serum blood glucose by 56.08 and 54.00%, respectively, compared to the STZ-induced diabetic group, and exceeding that of the GLB treated group. This concomitantly associated with a substantial increase in serum insulin evaluated by 4.1 and 12.7%, respectively that reflects that the effectiveness of BIT and BIE in ameliorating hyperglycaemia could rely on other mechanisms other than induction of insulin release from the pancreatic cells, such as inhibition of *α*-amylase and *α*-glucosidase activity. It is worth noting that significant in vivo activity of BIT and BIE with respect to the in vitro results may rely upon the enhancement of some metabolites by the physiological pH. 

##### Determination of the Oxidative Stress Markers

**Figure 2:**
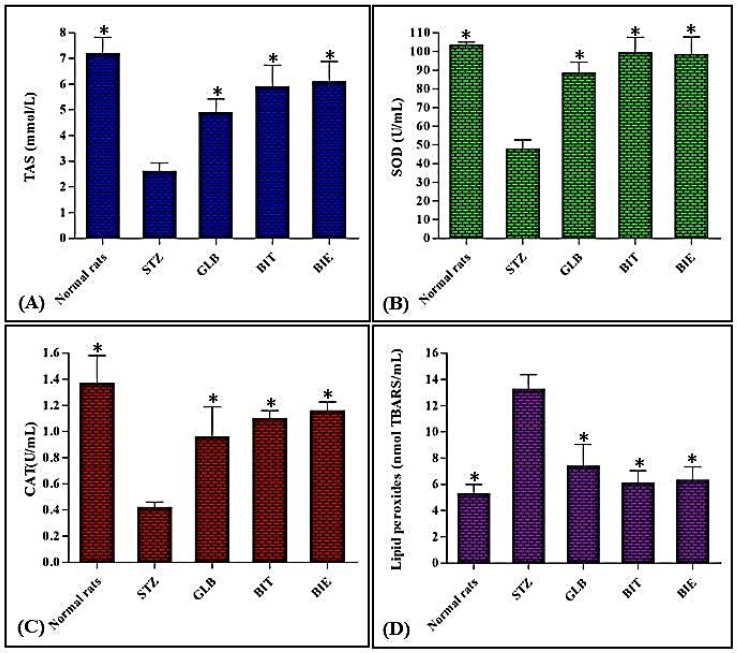
Effect of BIT and BIE oral administration on the oxidative stress markers; TAS (**A**), SOD (**B**), CAT (**C**) and lipid peroxides (**D**) in STZ-diabetic treated rats. Data are presented as means ± S.E.M. (measured in triplicates; *n* = 3), * significantly different from STZ at *p* < 0.05.

Regarding the oxidative stress markers, intraperitoneal administration of STZ causes a tremendous elevation in lipid peroxides, estimated by 149.33% relative to the normal untreated group. Additionally, this is associated by a considerable decrease in the levels of TAS, CAT and SOD by 62.57, 68.14 and 52.69%, respectively, in comparison to the normal untreated group. In contrast, oral administration of GLB significantly reduces lipid peroxides levels by 44.19% with concomitant elevation of TAS, CAT and SOD by 87.74, 128.57 and 85.11%, respectively, compared to the STZ-induced diabetic group. Meanwhile BIT and BIE displayed potent antioxidant activity evidenced by ameliorating the oxidative stress markers in a manner exceeding that of GLB. This is reflected in reducing lipid peroxides by both BIT and BIE by values equal to 54.00 and 52.41%, respectively, and this is concomitantly associated with a pronounced elevation in the levels of TAS by 126.44 and 133.72%, SOD by 107.49 and 105.61% and CAT by 161.90 and 176.19%, respectively (Figure 2A–D). It is clearly obvious that both BIT and BIE exerted potent antioxidant activity in addition to effectively ameliorating hyperglycaemia via reducing FBG that is mainly relied upon for their richness with phytoconstituents. A few *Buddleia* species were previously reported to possess antihyperglycaemic activity, such as *B. incana*, *B.officinalis*, and *B. polystachya* [15,16,17]. Although *B. cordata* was previously reported to contain linarin, vanilic acid and verbascoside, nothing was reported about its antihyperglycaemic activity. Meanwhile the different extracts of *B. polystachya* displayed potent antihyperglycaemic activity, particularly the ethyl acetate fraction followed by the dichloromethane fraction, whereas the *n*-butanol fraction revealed the least anti-hyperglycaemic potential that further consolidates our obtained results [17]. It is worth noting that *B. saligna*, a South African medicinal plant, is traditionally popular by its antidiabetic activity owing to *α*-glucosidase’s inhibitory potential of its triterpenes [18]. 

### 2.2. Major Phytoconstituents Present in Buddleia indica Leaves

**Figure 3:**
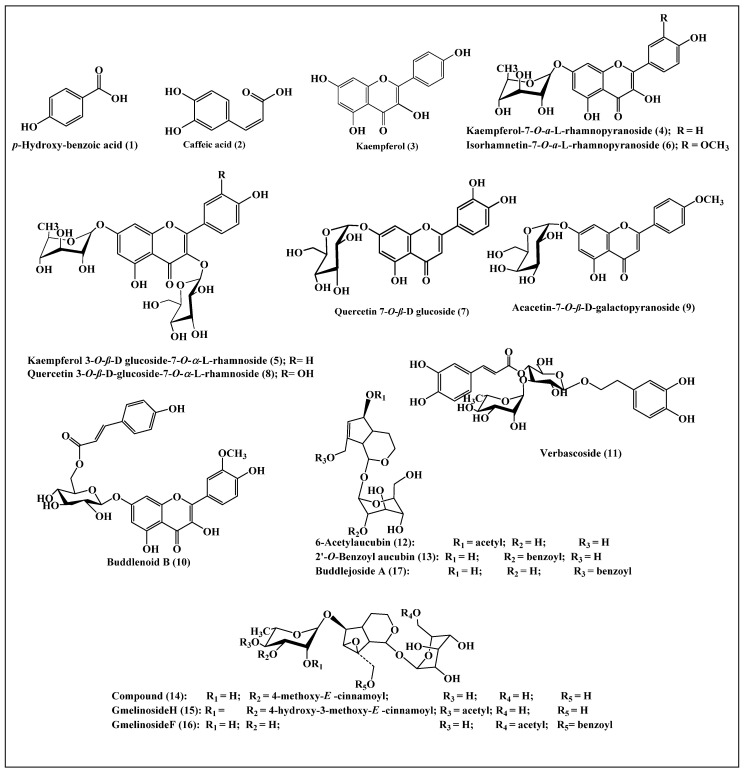
Scheme showing the major phytoconstituents present in *Buddleia indica* leaves.

*Buddleia indica* leaves are rich in polyphenolic compounds comprising flavonoids, phenolic acids, phenyl propanoids as well as iridoids. Several compounds have been previously reported by the authors with regard to the *Buddleia indica* leaves total methanol extract, which are *p*-hydroxy-benzoic acid, kaempferol-7-*O*-*α*-L-rhamnopyranoside, 6-acetylaucubin, gmelinoside H, catalpol 6-*O*-[4-methoxy-E-cinnamoyl-(3)-*α*-L-rhamnopyranoside, buddlenoid B, gmelinoside F, isorhamnetin 7-*O*-*α*-L-rhamnopyranoside. verbascoside, acacetin-7-galactoside, buddlejoside A and 2′-*O*-benzoyl aucubin using LC-ESI-MS [8]. Besides, *p*-hydroxy benzoic acid, caffeic acid, quercetin 3-*O*-*β*-D-glucoside-7-*O*-*α*-L-rhamnoside, kaempferol 3-*O*-*β*-D-glucoside-7-*O*-*α*-L-rhamnoside, quercetin 7-*O*-*β*-D glucoside, and kaempferol were isolated and structurally elucidated from the ethyl acetate fraction of *Buddleia indica* leaves total methanol extract using different chromatographic and spectroscopic techniques [9]. A scheme showing the major phytoconstituents present in *Buddleia indica* leaves, which are *p*-hydroxy benzoic acid (**1**), caffeic acid (**2**), kaempferol (**3**), kaempferol-7-*O*-*α*-L-rhamnopyranoside (**4**), kaempferol 3-*O*-*β*-D-glucoside-7-*O*-*α*-L-rhamnoside (**5**), isorhamnetin 7-*O*-*α*-L-rhamnopyranoside (**6**), quercetin 7-*O*-*β*-D-glucoside (**7**), quercetin 3-*O*-*β*-Dglucoside-7-*O*-*α*-L-rhamnoside (**8**), acacetin-7-galactoside (**9**), buddlenoid B (**10**), verbascoside (**11**), 6-acetylaucubin (**12**), 2′-*O*-benzoyl aucubin (**13**), catalpol 6-*O*-[4-methoxy-E-cinnamoyl-(3)-*α*-L-rhamnopyranoside (**14**), gmelinoside H (**15**), gmelinoside F (**16**), and buddlejoside A (**17**) is illustrated in Figure 3.

### 2.3. In Silico Molecular Modeling Studies

#### 2.3.1. Molecular Docking

In silico molecular docking studies were performed within the active sites of human *α*-amylase and human *α*-glucosidase for compounds previously isolated from the ethyl acetate fraction of *B. indica* Lam. leaves methanol extract as well as compounds identified from the total methanol extract to further confirm the obtained in vivo and in vitro results (Table 3).

**Figure 4:**
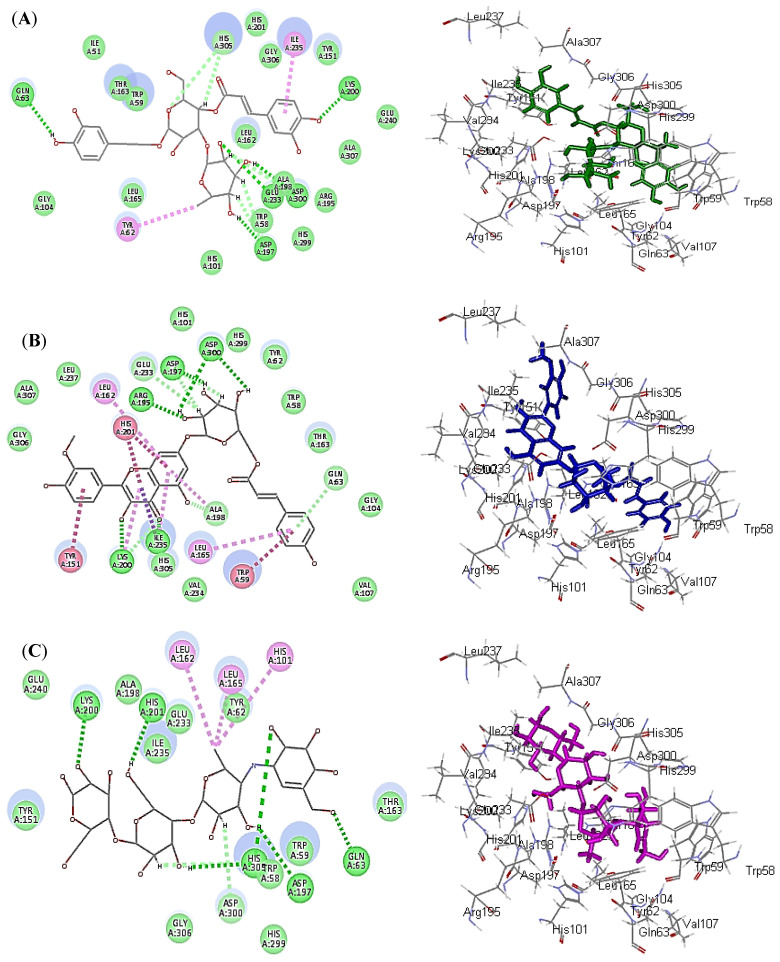
2D and 3D binding modes of verbascoside (**A**), buddlenoid B (**B**) and acarbose (**C**) within the active sites of human *α*-amylase (HA); heavy green dotted bond, H-bonds; heavy pink dotted bond, π-π bonds; light green dotted bond, C-H bonds; light pink dotted bond, π-alkyl bonds; purple dotted bond, π-δ bond.

Regarding human *α*-amylase inhibitory potential, verbascoside showed the highest fitting within the active sites followed by Buddlenoid B, displaying binding energies (∆G) of −33.33 and −31.49 Kcal/mole, respectively, approaching that of acarbose, the standard antihyperglycaemic agent (∆G = −37.50 Kcal/mole). The firm binding of verbascoside and buddlenoid B within the active site is attributed to the formation of many bonds with the amino acid residues present at the active site. Verbascoside forms five conventional hydrogen bonds with Asp197, Lys200, Glu233, Gln63 and Ala198; three C-H bonds with Trp58 and His305 in addition to the formation of two π-alkyl bonds with Ttyr62 and Ile235 (Figure 4A).

Meanwhile, buddlenoid B forms five conventional hydrogen bonds with Asp197, Asp300, Lys200, Ile235 and Arg195; four π-π bonds with His201, Trp59 and Tyr151; five π-alkyl bonds with Leu162, Lys200, Leu165, Ile235 and Ala198; two C-H bonds with Glu233 and Ala198; and one π-δ bond with Ile235 (Figure 4B). However, acarbose forms six conventional hydrogen bonds with His201, Asp197, Lys200, His305 and Gln63; two C-H bonds with Glu233 and Ala198; and three π-alkyl bonds with Leu162, Leu165 and His101 (Figure 4C).

**Figure 5:**
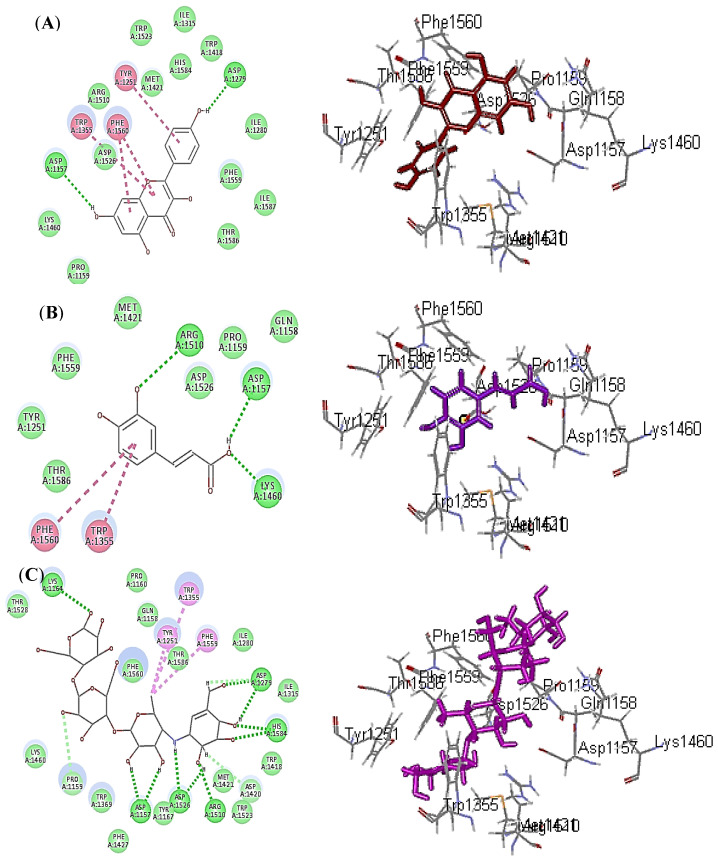
2D and 3D binding modes of kaempferol (**A**), caffeic acid (**B**) and acarbose (**C**) within the active sites of human *α*-glucosidase (HG); heavy green dotted bond, H-bonds; heavy pink dotted bond, π-π bonds; light green dotted bond, C-H bonds; light pink dotted bond, π-alkyl bonds.

Regarding human *α*-glucosidase, kaempferol showed the best fitting inhibitory potential, followed by caffeic acid with ∆G equal −33.06 and 30.44 Kcal/mole, respectively, approaching that of acarbose (∆G = −39.79 Kcal/mole). Kaempferol forms two conventional hydrogen bonds with Asp1279 and Asp1157 in addition to three π-π bonds with Trp1355, Phe1560 and Tyr1251 (Figure 5A).

However, caffeic acid forms three conventional hydrogen bonds with Asp1157, Lys1460 and Arg1510; two π-π bonds with Phe1560 and Trp1355 (Figure 5B). Meanwhile acarbose forms ten conventional hydrogen bonds with Asp1526, Asp1279, Asp1157, Lys1164, His1584 and Arg1510; two C-H bonds Pro1159 and Met1421; three π-alkyl bonds with Tyr1251, Phe1559 and Trp1355 (Figure 5C). In addition, all of these compounds form Van der Waals interactions with most of the amino acid moieties present at the active site of both enzymes. 

It is noteworthy to highlight that verbascoside, one of the major phytoconstituents in *B. indica*, displayed potent lens aldose reductase inhibitory potential with an IC_50_ of 3.1 μM. Additionally, it perfectly enhanced glucose tolerance and reduced glucose level at doses of 250–500 mg/kg in mice via prohibiting glucose transporter 1-mediated glucose uptake. Besides, it prohibited the accumulation of sorbitol in a rat lens after incubation with a high concentration of glucose by 70.6%. Furthermore, it significantly decreases blood glucose after oral administration with dosses of 10, 20, and 40 mg/kg to 111.30, 74.88, and 75.15 mg/dL, respectively, in diabetic rats with concomitant elevation of serum insulin level to be 3.23, 5.38, and 6.80 μU/mL, respectively, in diabetic rats [19]. In addition, kaempferol previously displayed potent in vitro *α*-glucosidase inhibitory potential in addition to improving glucose uptake in 3T3-L1 adipocyte [20,21]. Kaempferol significantly ameliorated hyperglycemia via acting at many targets. It could effectively enhance insulin-stimulated glucose uptake in mature 3T3-L1 adipocytes. Additionally, kaempferol acts as a weak partial agonist in the PPARγ reporter assay. However, it could not stimulate the differentiation of 3T3-L1 preadipocytes as commonly known PPARγ agonist meanwhile kaempferol could compete with rosiglitazone at the same binding pocket site as PPARγ. Besides, kaempferol displayed promising NO production generation after treatment of macrophage cells with lipopolysaccharide where PPARγ was overexpressed exceeding that of rosiglitazone. These observations suggest that kaempferol and quercetin potentially act at multiple targets to ameliorate hyperglycemia, including by acting as partial agonists of PPARγ [21].

#### 2.3.2. ADME/TOPKAT Prediction

**Figure 6:**
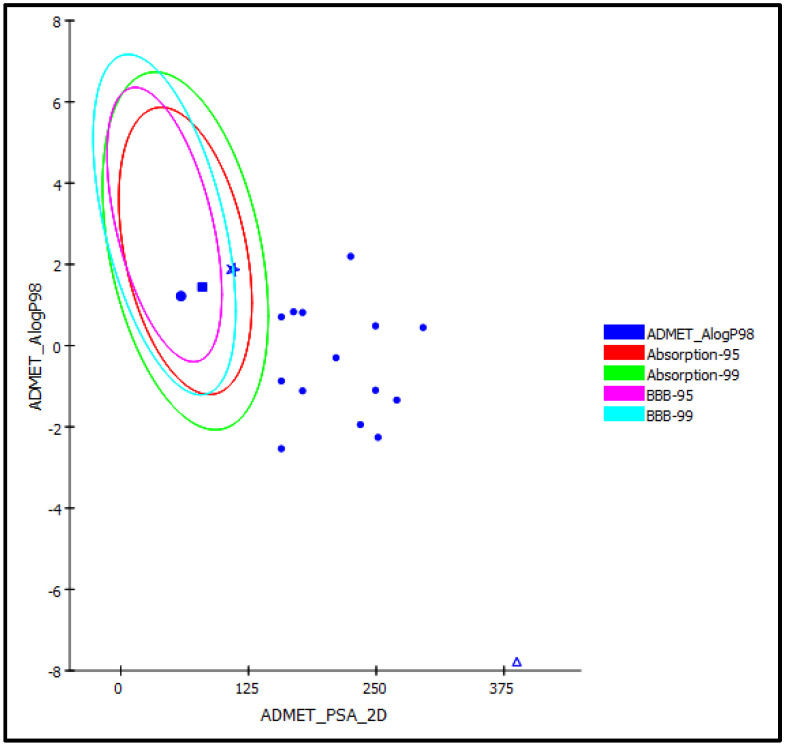
ADMET Plot for bioactive compounds from *Buddleia indica* leaves displaying 95% and 99% confidence limit ellipses corresponding to the blood-brain barrier (BBB) and the human intestinal absorption models; *p*-hydroxy benzoic acid (**1**) (large filled circle); caffeic acid (**2**) (filled square), kaempferol (**3**), (star); and acarbose (triangle) in ADMET_AlogP98.

ADME/TOPKAT (absorption, distribution, metabolism, excretion and toxicity) prediction was done for all the compounds isolated from the ethyl acetate fraction of *B. indica* Lam. leaves methanol extract as well as compounds previously identified from the total methanol extract to predict their pharmacokinetic, pharmacodynamic as well as toxicity properties. Results illustrated by ADMET plot (Figure 6) revealed that *p*-hydroxy benzoic acid (**1**), caffeic acid (**2**) and kaempferol (**3**) exhibited good human intestinal absorption and concomitantly lies in the 99% absorption ellipse. In contrast, all other compounds including acarbose displayed very low human intestinal absorption and thus present outside the 99% absorption ellipse in the ADMET plot. However, most of the examined compounds revealed optimal solubility except for quercetin 3-*O*-*β*-D-glucoside-7-*O*-*α*-L-rhamnoside (**8**) that displayed very low solubility and acarbose that showed extremely low solubility. Additionally, *p*-hydroxy benzoic acid (**1**), caffeic acid (**2**) and kaempferol (**3**) exhibited a low BBB (blood brain barrier) penetration level and hence lies inside the 99% BBB confidence eclipse in ADMET plot. In contrast, other compounds possess undefined BBB penetration level with level 4 and lies outside the 99% BBB confidence eclipse. Concerning PPB (plasma protein binding), all of the compounds showed 90% PPB. Additionally, all of the tested compounds displayed no inhibition to CYP2D6, meanwhile compounds (**1**) and (**3–9**) revealed certain hepatotoxic effects (Table 4). 

Concerning TOPKAT postulation, all of the tested compounds showed no mutagenicity except isorhamnetin 7-*O*-*α*-L-rhamnopyranoside (**6**) and quercetin 7-*O*-*β*-D-glucoside (**7**) that revealed certain mutagenicity. Moreover, all of the compounds revealed no carcinogenicity versus female rat (NTP) except for *p*-hydroxy benzoic acid (**1**) meanwhile isorhamnetin 7-*O*-*α*-L-rhamnopyranoside (**6**), quercetin 7-*O*-*β*-D-glucoside (**7**), quercetin 3-*O*-*β*-D-glucoside-7-*O*-*α*-L-rhamnoside (**8**) and buddlenoid B (**10**) displayed certain carcinogenicity versus male rat (NTP). However, the compounds showed rat oral LD50 values ranging between 0.36–10.57 g/kg-body-weight with buddlenoid B (**10**) and verbascoside (**11**) showed the highest LD50 of 3.45 and 10.57 g/kg-body-weight, respectively, whereas acarbose showed 53.41 /kg-body-weight as LD50. In a similar manner, all the examined compounds displayed rat chronic LOAEL levels ranging between 0.012 and 0.164 g/kg-body-weight where *p*-hydroxy benzoic acid (**1**) showed the highest value of 0.164 g/kg-body-weight. Furthermore, all of the examined compounds showed mild to no dermal irritation meanwhile most of the compounds showed mild to moderate eye irritation except *p*-hydroxy benzoic acid (**1**) that showed severe ocular irritation (Table 5). Thus, it can be concluded that verbascoside, caffeic acid, and kaempferol that showed highly potent antihyperglycaemic activity also possess reasonable pharmacokinetic, pharmacodynamic and toxicity properties and thus can be incorporated together with *B. indica* Lam. leaves in pharmaceutical preparations to alleviate diabetes.

## 3. Materials and Methods

### 3.1. Plant Material

*Buddleia indica* Lam. was brought from El-Orman Botanical Garden, Giza, Egypt, in 2019. The plant was morphologically identified and authenticated by the taxonomist Theresa Labib, Consultant of Plant Taxonomy at the Ministry of Agriculture, Giza, Egypt. A voucher number of PHG-P-BI-163 was given to the plant voucher specimen that was maintained at Pharmacognosy Department, Faculty of Pharmacy, Ain Shams University, Egypt.

### 3.2. Preparation of Buddleia indica Lam. Leaf Extract

500 g of *B. indica* Lam. air-dried leaves were coarsely powdered followed by maceration in 5 L of distilled methanol. This in turn was followed by filtration and this was done in repetition for three times until reaching the state of exhaustion. All the filtrates were collected and evaporated under vacuum at 45 °C to give semisolid that was followed by lyophilization to give about 120 g of total methanol extract (BIT). 105 g of BIT were dissolved in 70% methanol and subsequently fractionated using solvent-solvent fractionation by *n*-hexane, dichloromethane, and ethyl acetate in a successive manner to give 21.93, 8.32, and 27 g of each fraction, respectively, and 49.2 g remaining hydro-methanol fraction. 

### 3.3. Chemical Reagents and Kits for Biological Evaluation

Glibenclamide (GLB) and Streptozotocin (STZ) were obtained from Sigma Aldrich chemicals (St. Louis, MO, USA). Radioimmunoassay kit for the rat insulin was obtained from Amersham Biosciences; (Piscataway, NJ, USA). The glucose oxidase kit was purchased from Randox, UK. The Glucotest (glucose urine strips) was obtained from Roche Diagnostics Germany (Mannheim, Germany). Thiobarbituric acid for measurement of lipid peroxidation (LPO) was purchased from Fluka (Buchs, Switzerland). All remaining chemicals were of the highest grade and commercially available.

### 3.4. Biological Evaluation of the Anti-Hyperglycaemic Activity 

#### 3.4.1. In Vitro Anti-Hyperglycaemic Evaluation Using 3T3-L1 Adipocyte Culture

In vitro evaluation of the anti-hyperglycaemic using 3T3-L1 adipocyte culture was performed as previously described [22] in which 30 and 50 μg/mL of the tested sample stock solution dissolved in DMSO were used. The concentration of the DMSO does not exceed 0.1% in the working solution medium. Basically, insulin stock solution was done via solubilizing insulin (10^2^ M) in acetic acid (0.01 M; pH 3.0). Mouse pre-adipocytes 3T3-L1 cells (5 × 10^5^ cells/mL) from American Type Culture Collection (ATCC) were employed and subsequently cultured in DMEM medium enriched with 1% PS (penicillin–streptomycin) 5.56 mmol/L D-glucose and 10% FBS solutions. The cells were maintained in a humidified atmosphere at 37 °C with 5% CO_2_ and 95% air. 0.32 μM Insulin, 25 mmol/L of D-glucose, 1 μM dexamethasone and 0.5 mM 3-isobutyl-1-methylxanthine were used to treat the cells for 48 h after reaching 100% confluence. Untreated control 3T3-L1 cells, as well as those treated with BIT, were maintained for 24 h in DMEM media supplemented with 25 mmol/L of D-glucose. Insulin as well as pioglitazone were used as positive controls, whereas medium glucose concentrations were used to evaluate anti-hyperglycaemic potential of the examined sample [22].

#### 3.4.2. In Vitro Anti-Hyperglycaemic Evaluation Using Inhibition of *α*-Amylase Activity Assay

*α*-Amylase inhibitory potential was determined calorimetrically depending upon the method previously described by Funke et al. [23]. The enzyme reaction was determined using a microplate reader and monitored at 405 nm. The inhibition % of *α*-amylase was calculated using the following equation:

(1) % Inhibition=(Absorbance of the control−Absorbnce of the tested sample)×100

#### 3.4.3. In Vitro Anti-Hyperglycaemic Evaluation Using Inhibition of *α*-Glucosidase Activity Assay

*α*-Glucosidase inhibitory potential was determined calorimetrically depending upon the method previously described by Janibekov et al. [5]. A microplate reader adjusted at λ max = 405 nm was used to monitor *p*-nitrophenol absorbance. The inhibition % of *α*-glucosidase was calculated using the Equation (1).

#### 3.4.4. In Vivo Anti-Hyperglycemic Activity Assessment Using Streptozotocin Induced Hyperglycaemia in Rat 

##### Animals and Animal Treatment

130–220 g Wister male rats were obtained from King Abdulaziz University, Jeddah, Saudi Arabia. The study was conducted according to guidelines of the declaration of Helsinki. The experimental protocol gets approval from by the ethics committee at Faculty of Pharmacy, King Abdul-Aziz University, Jeddah, Saudi Arabia (Code # PH-1443-15). The animals were maintained at standard conditions of temperature and relative humidity estimated by 24 ± 5 °C for the former and 55 ± 5% for the latter in addition to a light/dark cycle of 12 h each. Animals were permitted to access standard laboratory water and pellets freely and accommodated to housing environmental conditions for one week before starting the study [24]. 

##### Induction of Hyperglycaemia in Rats

The induction of hyperglycaemia was achieved in rats via the administration of a single dose (60 mg/kg) of the hyperglycaemic agent, streptozotocin (STZ) intraperitoneal that was solubilized in citrate buffer (0.1 mL; pH 4.5). 5% Glucose in the drinking water was given to STZ-treated rats to avoid the occurrence of hypoglycemia that may happen initially; meanwhile vehicle only was used in the injection given to control animals. The presence of glucose in the urine of animals was monitored on the third day employing enzymatic test strips. Glibenclamide, standard oral hypoglycemic, was used as a positive control [1].

##### Experimental Protocol

The animals were grouped randomly into 5 groups each containing 8 rats. Normal control rats, group 1, administered only with citrate buffer. STZ-diabetic control animals, group 2, intraperitoneally injected with a single dose of 60 mg/kg of STZ. Group 3 is regarded as the positive control where STZ-diabetic rats were orally administered with 5 mg/kg b.wt of glibenclamide, GLB, for ten successive days. Group 4 and 5, the STZ-diabetic rats were orally administered with 50 mg/kg b.wt of BIT and BIE, respectively, for 10 successive days. The animals were exposed to light ether anesthesia followed by their scarification via cervical dislocation on the eleventh day. Heparinized chilled tubes with sodium fluoride were used in the collection of trunk blood. Centrifugation was used to separate the serum at 4 °C that is subsequently stored at −20 °C for the assessment serum insulin and serum glucose levels. Besides, oxidative stress was evaluated via the assessment of total antioxidant status (TAS), catalase (CAT) lipid peroxides (LPOs) and superoxide dismutase (SOD) as well.

##### Assessment of the Biochemical Parameters

(1) Assessment of Serum Glucose and Insulin Levels

Serum glucose was assessed using the glucose oxidase assay as previously reported by Trinder et al. respectively [2,25], meanwhile the insulin level was determined employing Amersham insulin immune reactive kit, as instructed by the manufacturer [26].

(2) Assessment of Serum Lipid Peroxides (LPOs)

This was estimated via the assessment of thiobarbituric acid reactive substances (TBARS) (nmol/mL). This was briefly done by mixing 5 mL serum homogenate with 1 mL of 6% TBARS in 0.25 M HCl and 1 mL of 1% H_3_PO_4_ that was subsequently heated for 50 min in boiling water bath. Cooling was done followed by the addition of 4 mL of *n*-butanol with continuous shaking. Centrifugation was done for 20 min at 6000 rpm/min to separate *n*-butanol layer then the difference between the absorbance at λ max = 535 and absorbance at λ max = 520 was determined [27].

(3) Assessment of Serum Total Antioxidant Status (TAS)

20 μL of the examined serum were added to a cuvette containing 1 mL chromogen that is prepared from 6.1 mmoL/L metmyoglobin and 610 mmoL/L 2,2′-azino-di-(ethylbenzthiazoline sulphonate). A1 (initial absorbance) was recorded then A2 was reported after three minutes from the addition of 200 μL of 250 mmoL/L H_2_O_2_, then the difference in the absorbance between the two readings was recorded [28] and TAS was calculated using the following equation:

TAS (mmol/L) = factor × (ΔA blank − ΔA sample)

Factor = Concentration of standard/(ΔA blank − ΔA standard)

(4) Assessment of Serum Catalase Activity

This was assessed by employing 2 mL of the reaction mixture containing hydrogen peroxide (1.95 mL of 10 mM in phosphate buffer (60 mM; pH 7). Initiation of the reaction was achieved via the addition of 0.1 mL of serum where the absorbance was determined after 3 min at 240 nm. The quantity of hydrogen peroxide changed to water and oxygen in 1 min under standard conditions is termed one CAT unit, and the specific activity was reported as U/mL [29].

(5) Assessment of Serum Super Oxide Dismutase Activity (SOD)

This was assessed using the xanthine oxidase method that depends on determining mitochondrial SOD activity of purified bovine erythrocyte SOD (5000 U/mg solid) that was used as the standard. Superoxide radicals as well as uric acid at pH 7.8 was produced via the reaction between 50 mM xanthine oxidase 1000 U, 50 mM xanthine and 1 mM EDTA (ethylene diamine tetra-acetic acid). 50 mM NBT reacted with the generated superoxide radicals to form red formazan dye that was determined spectrophotometrically at 250 nm. The existence of SOD in the analyzed sample (0.1 mL serum competes with the NBT for superoxide radical and prohibits the formation of formazan dye. U/mL was used in the expression of SOD activity [30].

##### Statistical Analysis

Statistical analysis was done employing one-way analysis of variance (ANOVA) where the results are expressed as mean ± standard error of the mean (SEM), *p* < 0.01 where additional comparisons were performed between groups employing post hoc Tukey’s test. GraphPad InStat 3 (GraphPad Software, Inc. La Jolla, CA, USA) software was used meanwhile GraphPad Prism version 5 software (GraphPad Software, Inc. La Jolla, CA, USA) was used to construct the graph.

### 3.5. In Silico Molecular Modeling Studies

#### 3.5.1. Molecular Docking

Molecular docking was done for all the compounds previously isolated from the ethyl acetate fraction of *B. indica* Lam. leaves methanol extract as well as compounds identified from the total methanol extract [8,9] employing Discovery Studio 4.5 (Accelrys Inc., San Diego, CA, USA) using C-docker protocol on human *α*-amylase (HA) (PDB ID 3BAY; 1.99 Å) and human *α*-glucosidase (HG) (PDB ID 3TOP; 2.88 Å), obtained from the protein data bank (www.pdb.org (accessed on 3 September 2021)) following what was previously reported [5,31].

#### 3.5.2. ADME/TOPKAT Prediction

ADME/TOPKAT (absorption, distribution, metabolism, excretion and toxicity) prediction was done for all the compounds isolated from the ethyl acetate fraction of *B. indica* Lam. leaves methanol extract as well as compounds previously identified from the total methanol extract [8,9]. Meanwhile, Ames mutagenicity, rat oral LD50, rat Chronic LOAEL, dermal and eye irritation as well as carcinogenic affect female and male rat NPT (National Toxicology Program) were selected as toxicity parameters. 

## 4. Conclusions

From this study it can be concluded that *Buddleia indica* leaves showed a promising antihyperglycaemic activity as demonstrated in vitro, in vivo, and in silico studies. This is attributed to the richness of *Buddleia indica* leaves with polyphenolic compounds comprising flavonoids, phenolic acids, phenyl propanoids, and well as iridoids. It is worth noting that verbascoside displayed the highest fitting within human *α*-amylase active sites whereas kaempferol showed the best fitting in human *α*-glucosidase active sites, approaching that of acarbose. Additionally, they displayed reasonable pharmacokinetic, pharmacodynamic and toxicity properties, as demonstrated by the ADME/TOPKAT prediction, and can thus be incorporated together with *B. indica* Lam. leaves in pharmaceutical preparations to alleviate diabetes. This would likely be welcomed by a large category of diabetes patients owing to the leaves’ natural origin. However, additional in vivo studies followed by clinical trials on the plant material and the identified compounds are highly recommended to validate its medicinal use as anti-hyperglycaemic agent. Furthermore, different pharmaceutical dosage forms containing *B. indica* Lam. leaves, either in the form of herbal teas or crude extracts, should be formulated after validation of its therapeutic potential.

## Figures and Tables

**Table 1:** Effect of *Buddleia indica* leaves total methanol extract (BIT) on glucose consumption in media of 3T3-L1 adipocyte cultures using 30 and 50 μg/mL.

	Glucose Concentration in mg/dL	
	30 μg/mL	50 μg/mL
Insulin	404.1 ± 3.9 *	388.0 ± 2.5 *
Pioglitazone	412.1 ± 4.1 *	379.8 ± 3.9 *
BIT	424.0 ± 3.8	389.2 ± 3.5 *
Control	430.7 ± 4.3	434.1 ± 3.6 *
Medium	455.0 ± 5.2	450.0 ± 4.3

Data are measured in triplicates (*n* = 3) and presented as means ± SEM. * Significantly different from the untreated control group at *p* < 0.05.

**Table 2:** Effect of BIT and BIE orally administered on serum glucose (mg/dL) and insulin (μU/mL) in STZ-diabetic rats.

	Group	Serum Glucose (mg/dL)	Serum Insulin (μU/mL)
1	Normal rats	119.35 ± 6.18 *	15.48 ± 1.35 *
2	STZ-diabetic rats	289.55 ± 8.03	9.68 ± 0.26
3	STZ-diabetic rats treated with GLB	154.60 ± 10.10 *	13.29 ± 1.89 *
4	STZ-diabetic rats treated with BIT	127.17 ± 12.81 *	10.08 ± 0.32
5	STZ-diabetic rats treated with BIE	133.20 ± 11.17 *	10.91 ± 0.36 *

Data are presented as means ± S.E.M. (measured in triplicates; *n* = 3), * significantly different from STZ at *p* < 0.05.

**Table 3:** Binding energies (kcal/mole) of major phytoconstituents present in *Buddleia indica* leaves within the active sites of human *α*-amylase (HA) and human *α*-glucosidase (HG).

Compounds	Human *α*-Amylase (HA)	Number of Formed Hydrogen Bonds with the Amino Acid Residues	Human *α*-Glucosidase (HG)	Number of Formed Hydrogen Bonds with the Amino Acid Residues
*p*-Hydroxy benzoic acid (**1**)	−21.50	3; Asp197, His101, His305	−24.48	2; Thr1586, Arg1510
Caffeic acid (**2**)	−28.04	2; Glu233, Asp300	−30.44	3; Asp1157, Lys1460, Arg1510
Kaempferol (**3**)	−27.92	3; Thr163, Glu233, Asp300	−33.06	2; Asp1279, Asp1157
Kaempferol-7-*O*-*α*-L-rhamnopyranoside (**4**)	−13.74	5; Glu233, Asp300, His299	−12.57	6; Trp1369, Asp1526, Arg1510, Asp1279, Lys1460, Asp1420
Kaempferol 3-*O*-*β*-D-glucoside-7-*O*-*α*-L-rhamnoside (**5**)	4.82	7; Thr163, Glu233, Gln63, His201, Asp197	6.37	4; His1584, Asp1279, Asp1157, Lys1460
Isorhamnetin 7-*O*-*α*-L-rhamnopyranoside (**6**)	−15.98	3; Gln63, Glu233, His201	−16.50	7; Asp1279, Asp1420, Lys1460, Arg1510, Trp1369
Quercetin 7-*O*-*β*-D-glucoside (**7**)	−17.81	5; Gln63, Tyr62, His101, Asp197, His201	−21.07	6; Asp1279, Asp1526, His1584, Arg1510
Quercetin 3-*O*-*β*-D-glucoside-7-*O*-*α*-L-rhamnoside (**8**)	−1.12	8; His101, Asp197, Arg195, Glu233, Lys200, His305	7.76	4; Asp1157, Asp1526, Lys1460
Acacetin-7-galactoside (**9**)	−15.20	3; Asp197, Lys200, Aala198	−15.96	2; Thr1586, Asp1526
Buddlenoid B (**10**)	−31.49	5; Asp197, Asp300, Lys200, Ile235, Arg195	−27.07	4; Asp1157, Gln1561, Lys1460
Verbascoside (**11**)	−33.33	5; Asp197, Lys200, Glu233, Gln63, Ala198	−25.82	9; Trp1369, Asp1526, Asp1279, Lys1460, Asp1157, Lys1164, Gln1158
6-Acetylaucubin (**12**)	22.89	4; Asp197, Glu233, His305	18.34	4; Trp1369, Lys1460, Asp1157
2′-*O*-Benzoyl aucubin (**13**)	15.27	1; His305	12.61	4; Arg1510, Lys1460, Asp1157
Catalpol 6-*O*-[4-methoxy-E-cinnamoyl-(3)-*α*-L-rhamnopyranoside (**14**)	3.95	2; Asp197, Glu233	3.43	7; Asp1279, Asp1526, Lys1460, Arg1510, Trp1369, His1584
Gmelinoside H (**15**)	FD	-	FD	-
Gmelinoside F (**16**)	6.35	1; Lys200	5.43	2; Lys1460, Asp1526
Buddlejoside A (**17**)	18.86	2; Thr163, Gly306	26.81	2; Lys1460, Asp1157
Acarbose	−37.50	6; His201, Asp197, Lys200, His305, Gln63	−39.79	10; Asp1526, Asp1279, Asp1157, Lys1164, His1584, Arg1510

Positive values indicate unfavorable interaction.

**Table 4:** Absorption, distribution, metabolism, excretion, and toxicity (ADMET) properties of major phytoconstituents present in *Buddleia indica* leaves.

Compounds	Absorption Level	Solubility Level	BBB Level	PPB Level	CPY2D6	Hepatotoxic	PSA-2D	Alog p98
*p*-Hydroxy benzoic acid (**1**)	0	4	3	False	NI	True	58.931	1.217
Caffeic acid (**2**)	0	4	3	False	NI	False	79.747	1.443
Kaempferol (**3**)	0	3	3	False	NI	True	109.492	1.872
Kaempferol-7-*O*-*α*-L-rhamnopyranoside (**4**)	3	3	4	False	NI	True	168.984	0.831
Kaempferol 3-*O*-*β*-D-glucoside-7-*O*-*α*-L-rhamnoside (**5**)	3	2	4	False	NI	True	249.29	−1.099
Isorhamnetin 7-*O*-*α*-L-rhamnopyranoside (**6**)	3	3	4	False	NI	True	177.914	0.814
Quercetin 7-*O*-*β*-D-glucoside (**7**)	3	3	4	False	NI	True	210.615	−0.299
Quercetin 3-*O*-*β*-D-glucoside-7-*O*-*α*-L-rhamnoside (**8**)	3	1	4	False	NI	True	270.106	−1.341
Acacetin-7-galactoside (**9**)	3	3	4	False	NI	True	157.098	0.706
Buddlenoid B (**10**)	3	2	4	False	NI	False	224.96	2.195
Verbascoside (**11**)	3	2	4	False	NI	False	249.29	0.484
6-Acetylaucubin (**12**)	3	5	4	False	NI	False	157.098	−2.537
2′-*O*-Benzoyl aucubin (**13**)	3	4	4	False	NI	False	157.098	−0.872
Catalpol 6-*O*-[4-methoxy-E-cinnamoyl-(3)-*α*-L-rhamnopyranoside (**14**)	3	3	4	False	NI	False	234.45	−1.945
Gmelinoside H (**15**)	3	3	4	False	NI	False	295.842	0.445
Gmelinoside F (**16**)	3	3	4	False	NI	False	251.75	−2.259
Buddlejoside A (**17**)	3	4	4	False	NI	False	177.914	−1.114
Acarbose	3	0	4	False	NI	False	387.553	−7.779

0, 1, 2, and 3 indicates good, moderate, low and very low absorption, respectively; 0, 1, 2, 3, 4, and 5 indicates extremely low, very low but possible, low, good, optimal, and too soluble, respectively; 0, 1, 2, 3, and 4 denote very high, high, medium, low, and undefined, penetration via BBB respectively. PBB, plasma protein binding, FALSE means less than 90%, TRUE means more than 90%. NI: Non-inhibitor. False: Non-toxic; True: Toxic.

**Table 5:** TOPKAT prediction of major phytoconstituents present in *Buddleia indica* leaves.

Compounds	Ames Prediction	Rat Oral LD50 g/kg_Body_Weight	Rat Chronic LOAEL g/kg_Body_Weight	Skin Irritancy	Ocular Irritancy	Rat Female NTP	Rat Male NTP
*p*-Hydroxy benzoic acid (**1**)	Non-Mutagen	1.37	0.164	None	Severe	Carcinogen	Non-Carcinogen
Caffeic acid (**2**)	Non-Mutagen	1.63	0.119	None	Moderate	Non-Carcinogen	Non-Carcinogen
Kaempferol (**3**)	Mutagen	0.96	0.148	None	Moderate	Non-Carcinogen	Carcinogen
Kaempferol-7-*O*-*α*-L-rhamnopyranoside (**4**)	Mutagen	1.92	0.079	None	Moderate	Non-Carcinogen	Carcinogen
Kaempferol 3-*O*-*β*-D-glucoside-7-*O*-*α*-L-rhamnoside (**5**)	Non-Mutagen	1.88	0.056	None	Moderate	Non-Carcinogen	Non-Carcinogen
Isorhamnetin 7-*O-α*-L-rhamnopyranoside (**6**)	Mutagen	1.90	0.061	None	Mild	Non-Carcinogen	Carcinogen
Quercetin 7-*O*-*β*-D-glucoside (**7**)	Mutagen	2.06	0.0587	None	Moderate	Non-Carcinogen	Carcinogen
Quercetin 3-*O*-*β*-D-glucoside-7-*O*-*α*-L-rhamnoside (**8**)	Non-Mutagen	2.27	0.074	None	Moderate	Non-Carcinogen	Carcinogen
Acacetin-7-galactoside (**9**)	Non-Mutagen	0.36	0.014	None	Mild	Non-Carcinogen	Non-Carcinogen
Buddlenoid B (**10**)	Non-Mutagen	3.45	0.016	Mild	Mild	Non-Carcinogen	Carcinogen
Verbascoside (**11**)	Non-Mutagen	10.57	0.095	Mild	Mild	Non-Carcinogen	Non-Carcinogen
6-Acetylaucubin (**12**)	Non-Mutagen	1.80	0.012	Mild	Mild	Non-Carcinogen	Non-Carcinogen
2′-*O*-Benzoyl aucubin (**13**)	Non-Mutagen	1.25	0.020	Mild	None	Non-Carcinogen	Non-Carcinogen
Catalpol 6-*O*-[4-methoxy-E-cinnamoyl-(3)-*α*-L-rhamnopyranoside (**14**)	Non-Mutagen	0.25	0.003	Mild	Mild	Non-Carcinogen	Non-Carcinogen
Gmelinoside H (**15**)	Non-Mutagen	0.57	0.005	Mild	Mild	Non-Carcinogen	Non-Carcinogen
Gmelinoside F (**16**)	Non-Mutagen	0.41	0.019	Mild	None	Non-Carcinogen	Non-Carcinogen
Buddlejoside A (**17**)	Non-Mutagen	0.80	0.023	Mild	None	Non-Carcinogen	Non-Carcinogen
Acarbose	Non-Mutagen	53.41	0.056	Mild	Severe	Non-Carcinogen	Non-Carcinogen

## Data Availability

Data are available upon request from the second author.
